# Sex-based analysis of first-line immune-based combinations in intermediate/poor-risk papillary, chromophobe, and unclassified renal cell carcinoma: an ARON-1 subanalysis

**DOI:** 10.3389/fonc.2026.1911444

**Published:** 2026-08-26

**Authors:** Paola I. Valdez-Sandoval, Evelyn L. Beas-Lozano, Zin W. Myint, Jakub Kucharz, Veronica Mollica, Alessandro Rizzo, Tarek Taha, Johannes Landmesser, Esther Pérez Calabuig, Jawaher Ansari, Cinzia Ortega, Ravindran Kanesvaran, Dipen Bhuva, Anca Zgura, Ondrej Fiala, Javier Molina-Cerrillo, Sebastiano Buti, Francesco Massari, Maria Fernanda Esparza-Orozco, Matteo Santoni, Maria T. Bourlon

**Affiliations:** 1 Instituto Nacional de Ciencias Médicas y Nutrición Salvador Zubirán, Mexico City, Mexico; 2 Division of Medical Oncology, Department of Internal Medicine, Markey Cancer Center, University of Kentucky, Lexington, KY, United States; 3 Department of Uro-Oncology, Maria Sklodowska-Curie National Research Institute of Oncology Warsaw, Warsaw, Poland; 4 Medical Oncology, IRCCS Azienda Ospedaliero-Universitaria Di Bologna, Bologna, Italy; 5 Struttura Semplice Dipartimentale (S.S.D.) Centro di Orientamento Oncologico (C.O.r.O.) Bed Management Presa in Carico, TDM, IRCCS Istituto Tumori “Giovanni Paolo II”, Bari, Italy; 6 Royal Marsden National Health Service (NHS) Foundation Trust, London, United Kingdom; 7 Clinic of Urology, Lübeck, Germany; 8 Medical Oncology Department, Complejo Hospitalario Universitario (CHU) Insular-Materno Infantil, Las Palmas de Gran Canaria, Spain; 9 Medical Oncology, Tawam Hospital, Al Ain, United Arab Emirates; 10 Division of Oncology, Institute for Cancer Research and Treatment, Asl Cn2, Alba-Brà, Italy; 11 Division of Medical Oncology, National Cancer Center Singapore, Singapore, Singapore; 12 Department of Medical Oncology, Army Hospital Research and Referral, New Delhi, India; 13 Department of Oncology-Radiotherapy, Prof. Dr. Alexandru Trestioreanu Institute of Oncology, “Carol Davila”, University of Medicine and Pharmacy, Bucharest, Romania; 14 Department of Oncology and Radiotherapeutics, Faculty of Medicine, University Hospital in Pilsen, Charles University, Pilsen, Czechia; 15 Biomedical Center, Faculty of Medicine in Pilsen, Charles University, Pilsen, Czechia; 16 ARON Research Foundation Ente del Terzo Settore (ETS), Macerata, Italy; 17 Department of Medical Oncology, Hospital Ramón y Cajal, Madrid, Spain; 18 Medical Oncology Unit, University Hospital of Parma, Parma, Italy; 19 Department of Medicine and Surgery, University of Parma, Parma, Italy; 20 Department of Medical and Surgical Sciences (DIMEC), University of Bologna, Bologna, Italy; 21 Medical Oncology Unit, Macerata Hospital, Macerata, Italy

**Keywords:** ARON-1, immune-based combination therapy, non clear cell renal cell carcinoma, real-world data, sex-based analysis

## Abstract

**Background:**

Non-clear cell renal cell carcinoma (nccRCC) represents a heterogeneous group of malignancies with limited prospective data to guide systemic therapy. Immune-based combination regimens are increasingly used in this setting by extrapolation from clear cell RCC trials. Whether patient sex influences outcomes in metastatic nccRCC treated with first-line immunotherapy (IO)-based combinations remains unknown.

**Methods:**

This is a retrospective sub-analysis of the ARON-1 real-world study that included 256 patients with intermediate- or poor-risk metastatic nccRCC treated with first-line IO-based combinations across 56 centers in 17 countries between January 2021 and December 2024. Patients with favorable-risk disease were excluded. The primary objective was to compare overall survival (OS) and time on treatment (ToT) between male and female patients. Tumor responses were assessed per RECIST 1.1 criteria. Survival outcomes were estimated using the Kaplan-Meier method and compared with the log-rank test. Cox proportional hazards regression models were used for univariable and multivariable analyses.

**Results:**

Of 256 patients included, 197 (77%) were male and 59 (23%) were female. The median OS in the overall population was 27.8 months (95% CI 22.6–36.5), with a 1-year OS rate of 80%. No statistically significant difference in OS was observed between females and males (31.9 months vs 26.7 months; p = 0.124). In the intermediate-risk subgroup, females demonstrated a longer median OS compared with males (36.1 vs. 27.8 months; HR 0.60; p = 0.041). Among patients with liver metastases, median OS was not reached in females versus 15.0 months in males (1-year OS: 88% vs. 53%; p < 0.001). In patients with papillary histology, females showed a significantly longer OS than males (36.1 vs. 27.8 months; HR 0.52; p = 0.037).

**Conclusions:**

In this large real-world study of IO-based combinations in metastatic nccRCC, no statistically significant sex-based difference in OS was observed in the overall population. Exploratory subgroup analyses identified potential sex-based interactions in intermediate-risk disease, liver metastases, and papillary histology that warrant prospective investigation. The underrepresentation of women in this cohort and the retrospective design underscore the need for dedicated, sex-stratified analyses in future trials.

## Introduction

Renal cell carcinoma (RCC) accounts for approximately 2–3% of adult malignancies worldwide. Among RCC, clear cell RCC (ccRCC) represents the most common histologic subtype, comprising nearly 70–80% of cases, whereas non-clear cell RCC (nccRCC) accounts for the remaining 20–30% and includes papillary, chromophobe, and unclassified tumors (1, 2). RCC is more frequent in men than women, with an incidence approximately twice as high in men (3, 4). This sex disparity is observed in both ccRCC and nccRCC; however, certain non–clear cell subtypes, such as chromophobe RCC, occur more commonly in women, a finding that may reflect underlying biological differences in tumor development between sexes (5, 6).

Compared to ccRCC, nccRCC has historically been associated with poorer outcomes, likely related to its lower incidence, its biological heterogeneity and the limited prospective data evaluating systemic therapies in advanced disease (7, 8). Pivotal phase III trials that established the current standard first-line therapy for RCC excluded nccRCC (9–12). As a result, the use of immune-based combination therapies in nccRCC has largely relied on extrapolation, complemented by evidence derived from small phase II studies (13–15). These evidence gaps make the management of advanced nccRCC particularly challenging, leaving clinicians to rely on data for ccRCC.

The efficacy of immunotherapy (IO) may differ between men and women as a result of biological, hormonal and environmental factors that can modify the immunological system. Women consistently mount more robust innate and adaptive immune responses, a phenomenon reflected in better response to vaccination, more efficient clearance of infectious agents, and a higher predisposition to autoimmune diseases (16, 17). These differences are largely mediated by sex hormones, particularly estrogens, as well as by genetic factors linked to the X chromosome, which influence antigen presentation, T-cell activation, and cytokine production (16, 18). In the oncologic setting, this heightened immune surveillance may paradoxically drive stronger immune-evasion mechanisms in tumors that ultimately progress. Consequently, tumors that progress to advanced stages in women may exhibit a more immunosuppressive tumor microenvironment and reduced immunogenicity (19, 20), leading to a decreased effectiveness of IO.

Non-biological factors may also contribute to sex-based differences in outcomes. Women have been historically underrepresented in clinical trials due to multiple barriers, including stricter eligibility criteria related to reproductive potential (9–12), caregiving responsibilities, and disparities in access to specialized care. Collectively, these factors may lead to delayed diagnosis, differences in treatment exposure, and ultimately impact survival outcomes.

Several meta-analyses have examined the influence of sex on IO efficacy, with heterogeneous results. Some analyses have suggested a greater relative benefit from IO in men (19, 21), whereas others have failed to demonstrate significant sex-based differences in survival outcomes (20, 22). In renal cell carcinoma specifically, evidence regarding sex-based differences remains limited. A previously published sub-analysis of the ARON-1 study found no significant sex-based differences in outcomes in the overall RCC population, although improved survival was observed in younger male patients and in men with clear cell histology (23).

Yet, whether patient sex influences outcomes in patients with nccRCC has not been systematically examined. Here, we report a retrospective sub-analysis of the ARON-1 real-world study, examining whether patient sex affects survival outcomes in patients with metastatic nccRCC receiving first-line immune-based combination therapies.

## Patients and methods

### Study population

This study involved a retrospective review of adult patients (aged 18 and older) diagnosed with metastatic non-clear cell renal cell carcinoma (nccRCC), confirmed either through histopathology or imaging studies. Only patients classified as intermediate- or poor-risk according to the International Metastatic RCC Database Consortium (IMDC) criteria at treatment initiation were included. Data were collected from patients treated with first-line immune-based combination therapies between January 1, 2021, and December 1, 2024, at 56 treatment centers across 17 countries (see **Supplementary Figure 1**).

First-line therapies were continued until one of the following occurred: disease progression (clinical or radiographic), unacceptable toxicity, or patient death. Follow-up imaging, including contrast-enhanced CT or MRI, was carried out every 8 to 12 weeks according to institutional guidelines. Routine physical examinations and laboratory evaluations were conducted every 4 to 6 weeks, consistent with local clinical practice.

Patient data were retrospectively extracted from medical records—both electronic and paper—and included demographics, tumor histology, International Metastatic RCC Database Consortium (IMDC) risk classification, prior nephrectomy status, metastatic sites, treatment-emergent adverse events, response to therapy, and subsequent lines of treatment. Patients were excluded if their tumor assessments were incomplete or if they were lost to follow-up.

### Study endpoints

This sub-analysis of the ARON-1 study aimed to evaluate sex-based differences in the real-world effectiveness of first-line immune-based combination treatments in patients with metastatic nccRCC, comparing outcomes between men and women.

Tumor responses were assessed locally using RECIST 1.1 criteria (24), and categorized into complete response (CR), partial response (PR), stable disease (SD), or progressive disease (PD). Objective response rate (ORR) was defined as the proportion of patients achieving CR or PR.

Overall survival (OS) was measured from treatment initiation to death from any cause. Time on Treatment (ToT) was calculated from the start of therapy to either disease progression or death. Patients who had not experienced disease progression or had started second-line treatments—or were lost to follow-up—were censored at the date of their last recorded assessment.

### Statistical analysis

Data analysis was performed using MedCalc version 19.6.4 (MedCalc Software, Broekstraat 52, 9030 Mariakerke, Belgium). All efficacy analyses were conducted to evaluate differences between male and female patients. OS and ToT were analyzed using Kaplan-Meier survival curves, with 95% confidence intervals (CIs) estimated by Rothman’s method. Survival outcomes were compared between men and women using the log-rank test. Cox proportional hazards regression models were used to assess the association between sex and survival outcomes. Sex (female vs male) was included as the primary variable of interest in both univariable and multivariable analyses. Multivariable models were adjusted for clinically relevant covariates, including age, prior nephrectomy, sarcomatoid differentiation, IMDC risk group, presence of bone, brain, liver, or lung metastases, and type of first-line treatment. Categorical variables were compared between men and women using the chi-square test or Fisher’s exact test, where appropriate. A p-value of less than 0.05 (two-tailed) was considered statistically significant.

## Results

### Patient population

Out of the 2,401 individuals included in the ARON-1 dataset who received immune-based combination therapies, 256 patients (representing 11%) had non-clear cell renal cell carcinoma (nccRCC) and were included in this analysis (**Supplementary Figure 2**). The median age of these patients was 63 years, with ages ranging from 23 to 84. The majority were male (197 patients, 77%), while females accounted for 23% (59 patients). Papillary histology was the most frequently observed subtype, identified in 148 patients (58%). Chromophobe histology was present in 36 individuals (14%), and 72 patients (28%) had tumors with unclassified histology (**Table 1**). Sarcomatoid features were noted in 49 cases (19%). A total of 157 patients (61%) had previously undergone nephrectomy. The lungs were the most frequently involved metastatic site, affecting 54% of patients. Based on the International Metastatic RCC Database Consortium (IMDC) risk classification, 190 patients (74%) were categorized as intermediate risk and 66 (26%) as poor risk. Regarding first-line treatment regimens, 76 patients (30%) were treated with IO+IO and 180 patients (70%) with IO+TKI (79 with pembrolizumab and axitinib, 69 with nivolumab plus cabozantinib, and 32 with pembrolizumab plus lenvatinib). A detailed breakdown of patient characteristics is provided in **Table 1**.

**Table 1:** Patient characteristics.

Characteristics	Overall no. (%)	Males	Females	*p*
Total patients	256 (100)	197 (77)	59 (23)	–
Age (years) Median (range)	63 (32–84)	63 (40–82)	65 (32–84)	–
ECOG-PS 0-1 ≥2	230 (90) 26 (10)	177 (90) 20 (10)	53 (90) 6 (10)	1.000
Tumor histology Papillary Chromophobe Unclassified	148 (58) 36 (14) 72 (28)	118 (60) 26 (13) 53 (27)	30 (51) 10 (17) 19 (32)	0.430
Sarcomatoid de-differentiation	49 (19)	35 (18)	14 (24)	0.386
Previous nephrectomy	157 (61)	119 (60)	38 (64)	0.662
Metastatic at diagnosis	148 (58)	116 (59)	32 (54)	0.568
Site of metastasis Lung Bone Liver Brain	138 (54) 78 (30) 61 (24) 7 (3)	107 (54) 62 (31) 41 (21) 5 (3)	31 (53) 16 (27) 20 (34) 2 (3)	1.000 0.538 0.057 1.000
IMDC Risk Group Intermediate Poor	190 (74) 66 (26)	145 (74) 52 (26)	45 (76) 14 (24)	0.870
First-line therapy IO+IO IO+TKI	76 (30) 180 (70)	62 (31) 135 (69)	24 (41) 35 (59)	0.145

### Survival analysis

**Figure 1:**
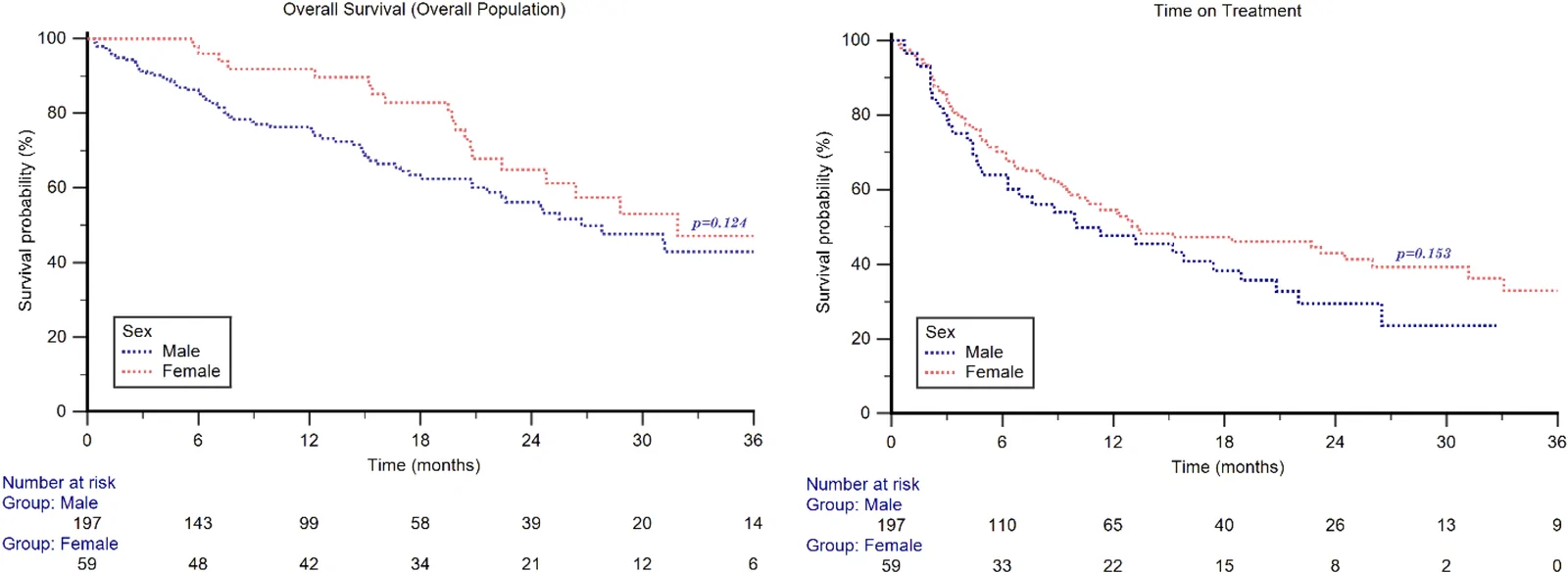
Overall survival and time on treatment in the overall study population.

The median follow-up time was 20.4 months (95%CI 16.6–111.4). The median OS in the overall study population was 27.8 months (95%CI 22.6–36.5), with a 1y-OS rate of 80%. The median OS was 31.9 months (95%CI 22.4–66.5) in females and 26.7 months (95%CI 21.6–40.5) in males (HR 0.71, 95%CI 0.45–1.10, *p* = 0.124, **Figure 1**), with a 1y-OS rate of 92% vs 76%, respectively (*p* = 0.003). Ninety-six patients (38%) were dead at time of data analysis, 75 males and 21 females.

The median OS was 28.8 months (95%CI 24.8–66.5) in patients with intermediate-risk features and 19.5 months (95%CI 7.6–56.8) in the poor-risk subgroup (HR 0.54, 95%CI 0.34–0.87, *p* = 0.011, **Supplementary Figure 3**). In the intermediate-risk subgroup, females reported a median OS of 36.1 months (95%CI 24.8–66.5), while males showed a median OS of 27.8 months (95%CI 22.4–65.3, HR 0.60, 95%CI 0.35–0.99, *p* = 0.041, **Supplementary Figure 3**). In patients with poor-risk features, no significant differences were found based on sex (females: 19.5 months, 95%CI 7.1–31.9; males: 15.0 months, 95%CI 7.0–56.8; HR 0.85, 95%CI 0.38–1.89, *p* = 0.684, **Supplementary Figure 3**).

We further stratified patients basing on the site of metastases. In patients with liver metastases, the median OS was not reached (NR) in females and 15.0 months (95%CI 6.1–24.6, HR 0.53, 95%CI 0.26–1.07, *p* = 0.057) in males, with a 1y-OS rate of 88% vs 53% (*p* < 0.001). No significant differences were found in patients with lung (females: 20.8 months, 95%CI 19.5–36.1; males: 24.6 months, 95%CI 20.7–62.3; HR 1.03, 95%CI 0.56–1.87, *p* = 0.927), or bone metastases (females: 28.8 months, 95%CI 15.4–66.5; males: 15.0 months, 95%CI 12.2–65.3; HR 0.55, 95%CI 0.27–1.15, *p* = 0.115).

Patients treated with nivolumab plus ipilimumab showed a median OS of 26.7 months (95%CI 19.5–66-5), being 36.1 months (95%CI 19.7–66.5) in females and 25.5 months (95%CI 12.2–26.7) in males (HR 0.76, 95%CI 0.38–1.52, *p* = 0.437). Otherwise, patients receiving Fine modulo.

IO plus TKI therapy reported a median OS of 27.8 months (95%CI 22.4–65.3), with median OS of 26.4 months (95%CI 20.8–31.9) and 27.8 months (95%CI 20.8–65.3) in females and males, respectively (HR 0.69, 95%CI 0.38–1.27 *p* = 0.236).

**Figure 2:**
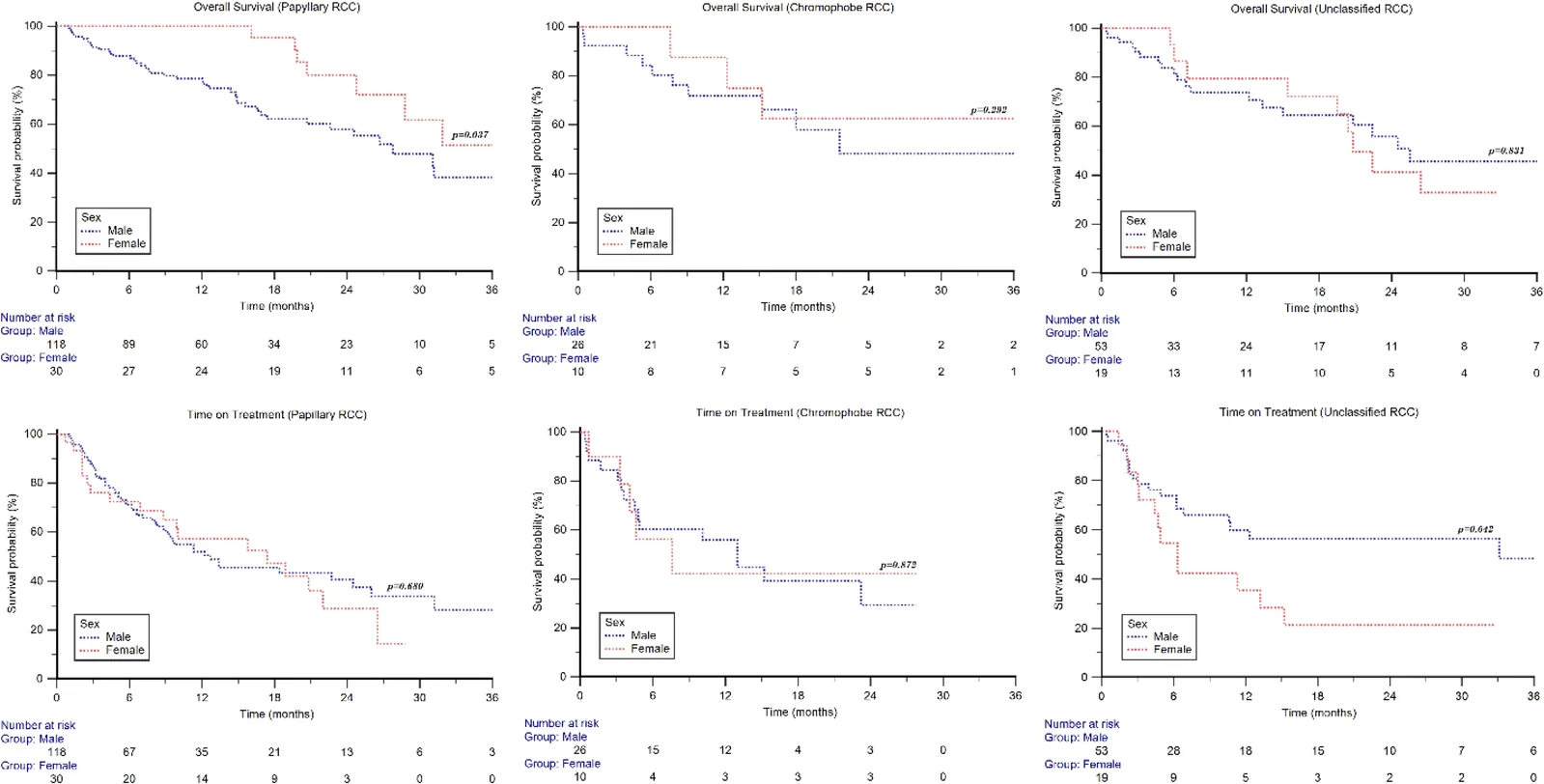
Overall survival and time on treatment in patients with papillary, chromophobe or unclassified renal cell carcinoma.

In patients with papillary RCC, the median OS was 31.1 months (95%CI 24.8–36.1) and was 36.1 months (95%CI 24.8–36.1) in females and 27.8 months (95%CI 20.7–65.3) in males (HR 0.52, 95%CI 0.28–0.96, *p* = 0.037, **Figure 2**).

In patients with chromophobe RCC, the median OS was 40.5 months (95%CI 7.6–66.5) and was NR in females and 21.6 months (95%CI 9.1–62.3) in males (HR 0.55, 95%CI 0.19–1.66, *p* = 0.292, **Figure 2**).

In patients with unclassified RCC, the median OS was 24.5 months (95%CI 19.5–56.8) and was 20.8 months (95%CI 7.1–26.4) in females and 25.5 months (95%CI 15.0–56.8) in males (HR 1.09, 95%CI 0.49–2.46, *p* = 0.831, **Figure 2**).

In the overall study population, nephrectomy, bone and liver metastases were significantly associated with OS at multivariable analysis (**Table 2**), while in patients with papillary RCC, sex, surgery and liver metastases were predictors of OS (**Table 3**).

**Table 2:** Univariable and multivariable analyses for overall survival in the study population.

Overall survival (overall population)	Univariable Cox regression		Multivariable Cox regression	
	HR (95%CI)	*p-value*	HR (95%CI)	*p-value*
Sex (females vs males)	0.68 (0.42−1.11)	0.127		
Age (>=70y vs <70y)	1.05 (0.56−1.79)	0.850		
Nephrectomy (yes vs no)	0.46 (0.31−0.70)	**<0.001**	0.52 (0.34−0.80)	**0.003**
Sarcomatoid differentiation (yes vs no)	0.97 (0.58−1.61)	0.894		
IMDC group (poor vs intermediate)	1.72 (1.12−2.63)	**0.012**	1.27 (0.81−2.00)	0.302
Lung metastases (yes vs no)	1.31 (0.87−1.98)	0.192		
Distant lymph node metastases (yes vs no)	1.26 (0.92−1.59)	0.179		
Bone metastases (yes vs no)	1.62 (1.07-2.45)	**0.022**	1.61 (1.06-2.45)	**0.024**
Liver metastases (yes vs no)	1.98 (1.29−3.04)	**0.002**	1.86 (1.18−2.92)	**0.007**
Brain metastases (yes vs no)	1.09 (0.40−2.97)	0.874		
First-line therapy (IO-IO vs IO+TKI)	0.93 (0.60−1.42)	0.723		

IMDC, International Metastatic RCC Database Consortium.The bold values indicate statistically significant results, defined as p-values < 0.05.

**Table 3:** Univariable and multivariable analyses for overall survival in patients with papillary RCC.

Overall survival (papillary RCC)	Univariable Cox regression		Multivariable Cox regression	
	HR (95%CI)	*p-value*	HR (95%CI)	*p-value*
Sex (females vs males)	0.45 (0.21−0.97)	**0.042**	0.39 (0.18−0.88)	**0.022**
Age (>=70y vs <70y)	1.04 (0.51−1.80)	0.735		
Nephrectomy (yes vs no)	0.41 (0.23−0.71)	**0.002**	0.46 (0.26−0.82)	**0.008**
Sarcomatoid differentiation (yes vs no)	1.08 (0.39−3.04)	0.879		
IMDC group (poor vs intermediate)	1.84 (0.96−3.54)	0.065		
Lung metastases (yes vs no)	1.08 (0.62−1.90)	0.785		
Distant lymph node metastases (yes vs no)	1.21 (0.91−2.04)	0.289		
Bone metastases (yes vs no)	1.83 (1.02-3.26)	**0.041**	1.52 (0.82-2.79)	0.182
Liver metastases (yes vs no)	2.46 (1.35−4.47)	**0.003**	2.79 (1.49−5.19)	**0.001**
Brain metastases (yes vs no)	1.46 (0.35−6.10)	0.597		
First-line therapy (IO-IO vs IO+TKI)	0.91 (0.48−1.72)	0.775		

IMDC = International Metastatic RCC Database Consortium.The bold values indicate statistically significant results, defined as p-values < 0.05.

### Time on treatment

The median ToT was 13.0 months (95%CI 10.0–20.8), with a 1y-ToT rate of 53%. The median ToT was 13.4 months (95%CI 10.1–26.0) in females and 10.0 months (95%CI 4.9–18.9) in males (HR 0.74, 95%CI 0.49–1.12, *p* = 0.153, **Figure 1**), with a 1y-ToT rate of 55% vs 47% (*p* = 0.263).

The median ToT was 15.2 months (95%CI 11.3–72.3) in patients with intermediate-risk features and 6.2 months (95%CI 3.6–15.2) in the poor-risk subgroup (HR 0.61, 95%CI 0.41–0.93, *p* = 0.021). In the intermediate-risk subgroup, females reported a median ToT of 18.4 months (95%CI 11.3–72.3), while males showed a median ToT of 11.3 months (95%CI 6.3–22.0, HR 0.66, 95%CI 0.40–1.09, *p* = 0.104). In patients with poor-risk features, no significant differences were found based on sex (females: 8.8 months, 95%CI 2.2–20.8; males: 6.2 months, 95%CI 3.2–22.7; HR 1.05, 95%CI 0.49–2.25, *p* = 0.902).

No differences in terms of median ToT were found in patients with liver (females: 6.2 months, 95%CI 3.1–13.4; males: 8.8 months, 95%CI 4.1–18.9; HR 1.08, 95%CI 0.56–2.09, *p* = 0.817), lung (females: 10.0 months, 95%CI 4.4–20.8; males: 13.4 months, 95%CI 9.3–26.0; HR 1.47, 95%CI 0.84–2.57, *p* = 0.173), or bone metastases (females: 4.7 months, 95%CI 2.1–15.2; males: 9.7 months, 95%CI 5.3–72.3; HR 1.24, 95%CI 0.61–2.49, *p* = 0.551).

Patients treated with nivolumab plus ipilimumab showed a median ToT of 6.8 months (95%CI 4.5–13.2), being 6.3 months in females (95%CI 2.1–13.2) and 8.3 months (95%CI 4.5–45.8) in males (HR 1.69, 95%CI 0.87–3.27, *p* = 0.123). Patients receiving Fine modulo.

IO plus TKI therapy reported a median ToT of 15.2 months (95%CI 12.2–72.3), with a median ToT of 17.4 months (95%CI 6.9–22.0) and 13.4 months (95%CI 11.3–72.3) in females and males, respectively (HR 1.10, 95%CI 0.65–1.87 *p* = 0.717).

In patients with papillary RCC, the median ToT was 13.4 months (95%CI 9.6–22.0) and was 17.4 months (95%CI 6.9–22.0) in females and 12.7 months (95%CI 9.0–72.3) in males (HR 0.89, 95%CI 0.51–1.55, *p* = 0.680, **Figure 2**).

In patients with chromophobe RCC, the median ToT was 13.0 months (95%CI 4.5–23.2) and was NR in females and 13.0 months (95%CI 4.5–23.2) in males (HR 0.92, 95%CI 0.34–2.51, *p* = 0.872, **Figure 2**).

In patients with unclassified RCC, the median ToT was 13.2 months (95%CI 6.3–33.1) and was 6.3 months (95%CI 3.1–15.2) in females and NR in males (HR 2.31, 95%CI 1.03–5.16, *p* = 0.042, **Figure 2**).

### Response to first-line therapy

In the overall study population, we observed 3% of CR, 35% of PR, 38% of SD and 24% of PD. In males we reported 2% CR, 36% PR, 39% SD and 23% PD, while females showed 5% CR, 34% PR, 36% SD and 25% PD.

**Figure 3:**
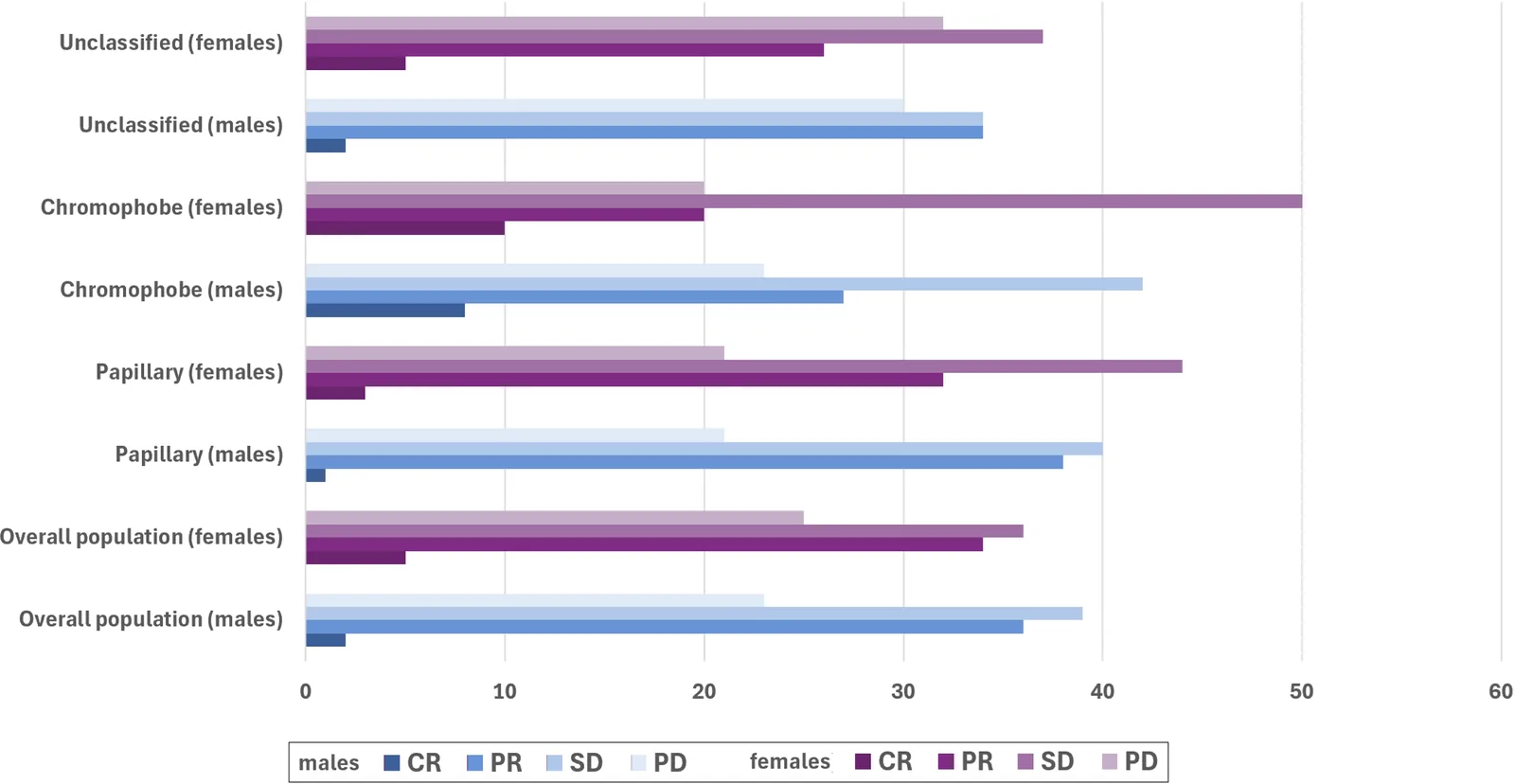
Response to first-line immune-combination therapy in patients with papillary, chromophobe or unclassified renal cell carcinoma.

Patients with papillary RCC showed 2% of CR, 39% PR, 38% SD and 21% PD. In this subgroup, males reported 1% CR, 38% PR, 40% SD and 21% PD, while in females we registered 3% CR, 32% PR, 44% SD and 21% PD (**Figure 3**).

In subjects with chromophobe RCC, we observed 8% of CR, 25% PR, 44% SD and 23% PD. Males showed 8% CR, 27% PR, 42% SD and 23% PD, while in females we registered 10% CR, 20% PR, 50% SD and 20% PD (**Figure 3**).

In subjects with unclassified RCC, we registered 3% CR, 32% PR, 35% SD and 30% PD. In this subgroup, males reported 2% CR, 34% PR, 34% SD and 30% PD, while in females we registered 5% CR, 26% PR, 37% SD and 32% PD (**Figure 3**).

## Discussion

This sub-analysis of the ARON-1 study is, to our knowledge, the largest real-world series evaluating IO-based combinations in metastatic nccRCC, and the first to specifically address sex-based differences in outcomes in this population. In this cohort of 256 patients, IO-based combinations demonstrated clinically meaningful activity, with a median OS 27.8 months in the overall population, further supporting their use as first-line therapy for advanced nccRCC in routine practice.

In this study, no statistically significant differences in overall survival (OS) were observed according to sex, with a median OS of 31.9 months in females as compared to 26.7 months in males (p = 0.124). The impact of sex on the efficacy of immunotherapy has been widely explored across tumor types, though findings have been inconsistent. A meta-analysis by Conforti et al. suggested a greater relative benefit of IO in men compared to women (pooled OS HR 0.72 vs. 0.86), whereas subsequent analyses, including Wallis et al., did not confirm a statistically significant difference between sexes (HR 0.75 vs. 0.77) (19, 22). In the specific context of advanced renal cell carcinoma (RCC), evidence remains limited and heterogeneous. Two systematic reviews that evaluated randomized clinical trials have demonstrated that IO confers an OS benefit irrespective of sex, without significant differences between males and females (25, 26). However, retrospective analyses of IO-based combinations have yielded heterogeneous findings. A previous report from the real-world ARON-1 study found no OS difference with first-line IO-based combination in the overall cohort (23), whereas two large-scale database analyses by Gordon et al. and Grigg et al. demonstrated worse OS and higher mortality in females with metastatic disease compared to males (27, 28). Importantly, studies demonstrating sex-based disparities have been predominantly conducted in cohorts enriched for clear cell histology, while analyses including nccRCC have generally not identified differences in OS according to sex (29), consistent with the findings of the present study.

Differences in outcomes between females and males in the IO era may be partially explained by previously described sex-based variations in the immune system. However, distinct biological features specific to RCC also contribute to these disparities. Among the biological factors implicated, hormonal signaling appears particularly relevant. Estrogen, acting through estrogen receptor beta, acts as a tumor suppressor factor by inhibiting RCC cell proliferation and invasion by downregulation of the AKT, ERK, and JAK pathways, which may partly explain the lower incidence of RCC in women (30). In the metastatic setting, estrogen can promote a pro-metastatic immune microenvironment in the liver through TNFR2-mediated accumulation of myeloid-derived suppressor cells (31). In contrast, androgen receptor signaling has also been implicated in RCC progression, enhancing hematogenous dissemination particularly to the lungs by upregulation of VEGF-A, while reducing lymphatic spread (32). Beyond hormonal influences, sex-specific genomic differences may further modulate disease behavior. The presence of X-linked tumor suppressor genes that escape X-inactivation (EXITS genes) has been proposed as a protective factor in women, potentially contributing to the lower incidence of RCC (33). However, once RCC develops, certain mutations appear to have sex-specific prognostic implications. For instance, mutations in BAP1 are more frequently observed in tumors from female patients and have been associated with worse survival outcomes, suggesting a potential mechanism underlying poorer prognosis in advanced disease (34, 35). These differences are also reflected in the tumor immune microenvironment. Male tumors are often characterized by a more “inflamed” phenotype with higher leukocyte infiltration, whereas tumors in advanced-stage female patients more frequently exhibit a “desert-like” microenvironment, with reduced infiltration of cytotoxic CD8+ T cells and natural killer T cells (29, 31). In our cohort, however, these molecular differences did not translate into a survival difference between sexes, a finding that may reflect the histological heterogeneity of our population, given that most of this biological evidence derives from ccRCC. The retrospective design of our study also introduces potential selection bias and unmeasured confounding that may have influenced outcomes.

Besides biology, disparities in cancer care between men and women deserve consideration when interpreting these results. In our study, women represented only 23% of the total population, consistent with their well-documented underrepresentation in randomized clinical trials in RCC and oncology overall. This partly reflects the lower incidence of RCC in women; however, prior analyses of trials supporting FDA-approved anticancer therapies have shown that women are underrepresented by approximately 15–20% relative to disease incidence (36), with underrepresentation observed in nearly two-thirds of trials (66%) (37). This disparity is particularly pronounced in kidney cancer, where female participation is significantly lower compared to disease burden (OR 0.63) (36). In addition to representation, disparities extend to treatment access and delivery. Large database analyses in metastatic RCC have demonstrated that female patients are less likely to receive systemic therapy and more likely to receive no treatment compared to males (38). Similarly, female sex has been identified as an independent factor associated with a lower likelihood of receiving systemic therapy, including immunotherapy, even after adjustment for clinical and sociodemographic variables (39). These disparities matter clinically, patients who do not receive systemic therapy have worse outcomes regardless of sex, and undertreatment of women may itself confound survival comparisons. The absence of significant OS differences between sexes in our cohort should therefore not be taken as evidence of true biological equivalence. Imbalances in representation and treatment exposure may be masking underlying differences that a larger or prospective study might detect.

While the overall OS analysis did not show a statistically significant difference between sexes, certain subgroup findings deserve attention, but they should be interpreted as hypothesis-generating given the exploratory nature of these analyses. In the intermediate-risk subgroup, females showed a longer median OS compared to males (36.1 vs. 27.8 months, HR 0.60, p = 0.041), a pattern not observed in the poor-risk group. Whether this reflects a true biological interaction between sex and IMDC risk category, or a coincidence in a relatively small subgroup, cannot be determined from the present data. A particularly striking observation was noted in patients with liver metastases, where the median OS was not reached in females compared to 15.0 months in males, with a 1-year OS rate of 88% versus 53% (p < 0.001). This finding is intriguing in light of preclinical evidence suggesting that estrogen-mediated accumulation of myeloid-derived suppressor cells in the liver may create a more immunosuppressive microenvironment (31), potentially driving worse outcomes in this specific metastatic context. However, the number of female patients with liver metastases in our cohort was small, and this observation requires prospective validation before any clinical conclusions can be drawn. Similarly, in patients with papillary histology, females showed a significantly longer OS than males (36.1 vs. 27.8 months, HR 0.52, p = 0.037), while no significant sex-based differences were observed in chromophobe or unclassified subtypes. The biological basis for this potential papillary-specific sex interaction is unclear, and again, these findings should be considered exploratory.

Several limitations of this study should be noted. First, the retrospective design introduces the potential for selection bias and unmeasured confounding. Differences in comorbidity burden, performance status, access to care, and exposure to subsequent lines of therapy between male and female patients were not fully captured and may have influenced survival outcomes. Second, the number of female patients was relatively small (n = 59), which limits the statistical power of sex-stratified analyses and the reliability of subgroup estimates. Third, the heterogeneity of nccRCC itself makes it difficult to draw uniform conclusions across subtypes. Fourth, treatment-related adverse events were not analyzed in the present manuscript. Although clinically relevant, these outcomes have been analyzed in a recent ARON-1 publication that included sex-based differences in toxicity profiles (40). Finally, patients with favorable-risk disease according to IMDC criteria were not included in this analysis. This decision was deliberate, since nivolumab plus ipilimumab, one of the four IO-based regimens evaluated, is not routinely used in favorable-risk patients in most centers, and its inclusion would have introduced an imbalance in treatment allocation across risk groups that could have confounded comparisons. By restricting the analysis to intermediate- and poor-risk patients, we aimed to ensure a more comparable treatment landscape across the cohort.

## Conclusion

In this large real-world study of IO-based combinations in metastatic nccRCC, no statistically significant difference in overall survival was observed between male and female patients. IO-based combinations demonstrated clinically meaningful activity regardless of sex, supporting their use as first-line therapy in intermediate- and poor-risk nccRCC in routine practice. Exploratory subgroup analyses suggest potential sex-based interactions in intermediate-risk disease, liver metastases, and papillary histology, though these findings are hypothesis-generating and require prospective validation. The underrepresentation of women in this cohort highlights the need for dedicated efforts to improve female enrollment in RCC trials and to conduct adequately powered, sex-stratified analyses in future studies.

## Data Availability

The raw data supporting the conclusions of this article will be made available by the authors, without undue reservation.

## Supplementary material

Supplementary Figure 1 List of participating Countries.

Supplementary Figure 2 Selection process from the ARON-1 dataset.

Supplementary Figure 3 Overall Survival by IMDC group and sex.
